# Functional Neurological Disorder Presenting as Apparent Drug-Resistant Seizures: A Case Report on Emphasizing the Role of Early Diagnosis

**DOI:** 10.7759/cureus.99613

**Published:** 2025-12-19

**Authors:** Emmanuel F Victoria Martinez, Carol Montserrat García Escalante, Irene Gómez-Oropeza, Daniela Deustúa-Hernández, Elma Paredes-Aragón

**Affiliations:** 1 Neurology, Benemérita Universidad Autónoma de Puebla, Puebla City, MEX; 2 Neurology, Instituto Nacional de Neurologia y Neurocirugia, Mexico City, MEX; 3 Neurosurgery, Instituto Nacional de Neurologia y Neurocirugia, Mexico City, MEX; 4 Medical Education, Benemérita Universidad Autónoma de Puebla, Puebla City, MEX; 5 Epilepsy, Instituto Nacional de Neurologia y Neurocirugia, Mexico City, MEX

**Keywords:** case report, epilepsy, functional neurological disorder, functional seizures, psychogenic non-epileptic seizure

## Abstract

Functional neurological disorder (FND) is a complex and frequently misdiagnosed condition characterized by neurological symptoms such as motor events, sensory deficits, or altered consciousness caused by brain network dysfunction rather than structural abnormalities. Despite its prevalence, FND remains stigmatized and underrecognized, mainly due to its historical association with psychological factors and its disproportionate impact on women. We present the case of a 19-year-old biological woman with a seven-year history of presumed epilepsy, treated with multiple antiseizure medications despite inconclusive findings on brain magnetic resonance imaging (MRI) and EEG. Her medical history included post-traumatic stress disorder and a family history of epilepsy involving both first and second degree relatives, including one case associated with tuberous sclerosis. At 18 years of age, she was hospitalized after several functional seizures requiring orotracheal intubation, though EEG and brain MRI remained normal. She continued to experience functional seizures. A video-EEG eventually recorded multiple events without an electrographic correlate, confirming the FND diagnosis. Following diagnosis, antiseizure medications (ASM) were gradually withdrawn, and she initiated cognitive-behavioral therapy and sertraline. The patient achieved complete resolution of functional events and reported significant improvement. This case highlights the importance of early consideration of FND in patients with drug-resistant seizures and non-correlating diagnostics to avoid unnecessary treatments and improve quality of life.

## Introduction

Functional neurological disorder (FND) is a complex and frequently misdiagnosed condition characterized by neurological symptoms, such as paroxysmal motor events or sensory deficits, whose severity or pattern cannot be fully explained by the underlying anatomical or physiological findings [1,2]. It is understood as a disorder of brain network dysfunction rather than of structural damage [2,3], and accounts for approximately 5-10% of new neurology outpatient consultations, making it nearly as prevalent as epilepsy [2]. Historically, FND has been poorly understood and widely stigmatized [2,4]; early interpretations attributed symptoms to uterine disturbances, giving rise to the term hysteria misconception that persisted for centuries and reinforced gender-biased views of the disorder [4-6]. Explanations later shifted toward emotional causes such as melancholia or hypochondriasis [4], until late 19th and early 20th century clinicians such as Charcot and Freud reframed the condition within emerging neurological and psychodynamic theories, exploring mechanisms like hypnosis and proposing that psychological conflicts could manifest as physical symptoms [2,4,7].

The evolution of psychiatric classification has reflected this shifting understanding [4]. The first edition of the Diagnostic and Statistical Manual of Mental Disorders (DSM) included FND-like presentations under psychosomatic disorders [8]. Over time, the term hysteria came to be viewed as imprecise, pejorative, and representative of diagnostic uncertainty [4,6]. It also became a source of social and clinical stigma, particularly for women and marginalized populations, often leading to dismissal of patient experiences and delays in appropriate care [2,4]. In the DSM-5, the condition was redefined as functional neurological symptom disorder, recognizing its neurobiological underpinnings and distancing it from outdated terminology [8]. According to the DSM-5, the diagnostic criteria for FND include the presence of one or more symptoms of altered voluntary motor or sensory function, clinical evidence of incompatibility between the symptoms and recognized neurological or medical conditions, exclusion of alternative medical or mental explanations, and the presence of clinically significant distress or functional impairment associated with the symptoms [8]. Importantly, several subtypes of FND have been described, including functional motor symptoms (such as weakness, tremor, dystonia, or gait disorders), functional sensory symptoms (such as anesthesia or visual loss), and functional seizures, also termed psychogenic non-epileptic seizures (PNES), which represent one of the most common and disabling manifestations of the disorder [9].

Socially, patients with FND frequently encounter substantial barriers to diagnosis, treatment, and acceptance within both healthcare systems and society at large [1,2]. Due to its historical associations, many patients report feeling disbelieved or dismissed by clinicians and family members [1,2,10]. This stigmatization often exacerbates psychiatric comorbidities and leads to social isolation, unemployment, and decreased quality of life [2]. The separation of organic and functional conditions in medicine continues to perpetuate misunderstanding and fragmented care [2]. FND can pose significant diagnostic and therapeutic challenges, often requiring careful consideration of clinical subtleties and psychosocial factors. In light of these complexities, we present this case to illustrate the multifaceted nature of FND and to emphasize the importance of integrated, informed, and empathetic approaches to its diagnosis and management.

## Case presentation

A 19-year-old ambidextrous woman with a prior diagnosis of epilepsy presented to our neurology department with multiple functional seizures exhibiting highly variable semiology (Table 1). Her past medical history was unremarkable for perinatal complications, head trauma, or CNS infections. She reported a history of sexual abuse during adolescence. Her family history included epilepsy in first and second degree relatives, one of whom had epilepsy associated with tuberous sclerosis.

**Table 1:** Clinical characteristics of eight functional seizures semiologies recorded in the patient, detailing ictal and post-ictal features. The table presents ictal semiology, the presence of long-duration events with asynchronous movements (LDEAM), loss of awareness, sphincter control, ictal duration, post-ictal manifestations, and event frequency. Most episodes did not evolve into LDEAM events, and awareness was frequently preserved. The characteristics of her events were initially described in detail by family members, who provided consistent eyewitness accounts throughout her evaluation. During hospitalization, additional episodes were directly observed and documented by the clinical team, allowing a more comprehensive characterization of her manifestations, which included focal motor features (clonic movements in semiology 3), sensory auras (an olfactory hallucination in semiology 6), and behavioral phenomena (inappropriate laughter in semiology 5). Event duration varied substantially, from seconds to several hours, and post-ictal symptoms ranged from headache to transient speech impairment. NA: not available; indicates that the information was not provided by the patient, witnesses, or medical records; LDEAM: long-duration event with asynchronous movements.

Event Type	Ictal Semiology	Conversion to LDEAM	Loss of Awareness	Loss of Sphincter Control	Ictal Duration	Post-Ictal Semiology	Post-Ictal Duration	Frequency
1	Disconnection from the environment	No	NA	No	15 seconds–2 minutes	Headache	The remainder of the day in question	10 times per month
2	Supraversive eye deviation	No	NA	No	15 seconds–2 minutes	Headache	Remainder of the day	10 times per month
3	Clonic movements of the right upper and lower limbs, independently and together	No	No	No	5 minutes-2 hours	Muscle pain and fatigue	10 times per year	NA
4	Repetitive and involuntary head turning to the right	No	No	No	Up to 30 minutes	NA	NA	NA
5	Inappropriate laughter	No	Yes	Yes	5 minutes	NA	No episodes in the past year	NA
6	Mint-odor aura followed by a prolonged episode with irregular, asynchronous limb movements	Yes	Yes	No	1 minute	Recovery of awareness with the inability to speak (up to 5 minutes)	2 hours	4-6 times within 24 hours during the emergency room stay in August 2024
7	Sudden disconnection from the environment with generalized tonic posture	NA	NA	NA	2-5 minutes	NA	NA	6 times per month, predominantly nocturnal
8	Fainting with generalized loss of strength, initially referred to as legs giving out.	No	NA	NA	NA	NA	NA	NA

She was initially diagnosed externally with epilepsy and dysautonomia seven years earlier, coinciding with her first menstrual period. Although she was labeled with epilepsy at that time, the events described retrospectively were consistent with non-epileptic episodes, as her EEG showed no abnormalities, and subsequent evaluations at our institution revealed no electrographic seizure activity. These early episodes were characterized by fever, nausea, visual disturbances including black and white spots, and pronounced polydipsia. Symptoms lasted approximately five minutes and were followed by a sudden loss of consciousness, described as a complete loss of postural tone. Medical evaluation occurred within three minutes, after which she exhibited a brief postictal state with confusion and remained somnolent for the rest of the day. A lumbar puncture was performed due to concern for autoimmune or inflammatory disease, and empirical immunotherapy was initiated.

At age 18, the patient experienced her first documented event with semiology consistent with a functional seizure, which progressed to a prolonged episode with asynchronous movements. The following day, she experienced six similar events within six hours, characterized by clonic movements of the right upper and lower extremities, occurring independently and simultaneously, but without loss of consciousness. These episodes prompted her admission to the emergency department of a private hospital. During that hospitalization, she was diagnosed with status epilepticus and developed peri-ictal hypoxia and perioral cyanosis, requiring orotracheal intubation. She was subsequently transferred to our institution for further management.

At our emergency department, sedation with midazolam was initiated, and the initial evaluation, including vital signs, neurological examination, laboratory studies with a normal serum lactate level, and cerebrospinal fluid analysis, was unremarkable. Continuous EEG monitoring was performed, and multiple clinical events were captured without any associated electrographic correlate (Appendix A). Although these findings raised suspicion for functional non-epileptic seizures, interpretation was initially cautious due to the recent external diagnosis of status epilepticus and the confounding effects of sedative medication. Nevertheless, throughout the sedation period, she exhibited recurrent motor events without returning to baseline, yet no EEG correlate was present, reinforcing early consideration of non-epileptic activity. She subsequently remained event-free for 48 hours and was successfully extubated. Bronchial cultures later grew *Staphylococcus aureus*, prompting intravenous vancomycin therapy.

One week later, after achieving hemodynamic and neurological stability, she was transferred to the neurology inpatient unit. Although the semiology and EEG suggested possible functional events, the use of three antiseizure medications reflected her initial working diagnosis of refractory status epilepticus, supported by external reports of epileptic activity and persistent motor events without baseline recovery. Her prior epilepsy further raised concern for potential comorbid seizures during the acute phase. Lamotrigine was considered but avoided due to a documented allergy and a prior episode of suspected arrhythmia. She improved on lacosamide, valproate, and brivaracetam, and after ten days was discharged on this regimen with biweekly follow-up at the epilepsy clinic.

At the epilepsy clinic, neuroimaging-including PET and a brain MRI with the harmonized neuroimaging of epilepsy structural sequences (HARNESS) protocol, showed no abnormalities. She later returned to our emergency department on two separate occasions, again exhibiting multiple events with variable semiology. During follow-up, she also reported new spatial and cognitive symptoms suggestive of right frontoparietal and temporal dysfunction. Given ongoing concerns, a video-EEG (VEEG) study was performed and captured six typical events without epileptiform activity, confirming the diagnosis of functional non-epileptic seizures in accordance with LaFrance’s clinical criteria [11]. This evaluation clarified that both her earlier childhood events and her recent episodes were non-epileptic in origin.

Two weeks after receiving the diagnosis of FND at our epilepsy clinic, she attended her first psychiatric consultation, where she was diagnosed with major depressive disorder and post-traumatic stress disorder (PTSD). She was started on sertraline at that time. During this period, the patient also began experiencing significant side effects from the ASM, including marked weight gain, likely due to valproate, and emotional lability. These symptoms, particularly intense sadness and unmanageable crying, were frequently noted during follow-up consultations and played a role in deciding to initiate psychiatric treatment.

Following the identification of ASMs' adverse effects and the revised diagnosis, a gradual discontinuation of anti-seizure therapy was carried out. Antiseizure medications were tapered off in sequence, with biweekly dose reductions. However, during the second month of withdrawal, the patient experienced multiple functional seizures, leading to a slower tapering schedule. Currently, clonazepam and lacosamide have been discontinued, and she remains on a low dose of valproic acid, which is being gradually reduced. She has been event-free, is undergoing cognitive-behavioral therapy (CBT), and reports an improvement in her emotional well-being.

## Discussion

FND is a condition that can closely resemble epilepsy in its clinical presentation, often leading to misdiagnosis [2]. It is commonly seen in neurological practice, with an estimated incidence of 19 to 22 cases per 100,000 people [7]. However, this number may vary significantly due to the high rate of misdiagnosis [5]. Studies show that 78% to 93% of individuals diagnosed with FND are women [8], and more than two-thirds also have psychiatric conditions [9]. Our patient, a 19-year-old woman with post-traumatic stress disorder, exemplifies both the demographic and clinical patterns most often reported, supporting existing epidemiological trends and fitting within the typical FND spectrum.

The etiology of FND involves many factors [1,2,8,11]. Significant risks include traumatic experiences and ongoing psychological stress [2]. Some research also suggests a link with epigenetic changes, such as increased methylation of the oxytocin receptor gene, which plays a key role in regulating the stress response system [7]. Other factors include a history of neurological disorders or adverse effects from long-term use of antiepileptic drugs [8]. In our patient’s case, all these factors are present: a history of sexual abuse, a diagnosis of post-traumatic stress disorder, and a seven-year history of epilepsy treated with various antiepileptic medications, exposing her to their cumulative side effects. These are better described in Table 2.

**Table 2:** Diagnostic criteria for functional neurological disorder. This table outlines the clinical criteria applied to establish the diagnosis of FND and functional crisis. In the present patient, all listed criteria were fulfilled, including seizure events recorded on video-EEG without epileptiform activity, characteristic semiology observed by experienced clinicians, absence of epileptiform activity on ambulatory and ictal EEG recordings, a detailed clinical history consistent with FND, normal neuroimaging findings, presence of psychosocial stressors or psychiatric comorbidities, and clinical improvement following psychological intervention or explanation of the diagnosis. These findings substantiate the diagnosis of FND in this case. FND: functional neurologic disorder; EEG: electroencephalography.

Criterion	Presence
Event captured on video-EEG with no epileptiform activity before, during, or after the event	Present
Typical semiology observed by an experienced clinician (in-person or video), compatible with PNES	Present
No epileptiform activity during ambulatory or ictal EEG despite expected EEG changes in true epilepsy	Present
Detailed history consistent with PNES, including fluctuating course, long duration, or stress-related	Present
Interictal EEG is normal or shows no epileptiform activity	Present
Normal brain imaging (MRI/CT)	Present
Eyewitness description or video of the event consistent with PNES, without EEG	Present
Event semiology includes closed eyes, asynchronous movements, and ictal crying, among others.	Present
History of psychosocial stressors, trauma, or psychiatric comorbidity	Present
Resolution or improvement after psychological intervention or after diagnosis is explained	Present
Event features inconsistent with epilepsy (e.g., prolonged duration, fluctuating, preserved awareness)	Present
One or more symptoms of altered voluntary motor or sensory function.	Present
Clinical findings provide evidence of incompatibility between the symptom and recognized neurological or medical conditions.	Present
The symptom or deficit is not better explained by another medical or mental disorder.	Present
The symptom or deficit causes clinically significant distress or impairment in social, occupational, or other important areas of functioning, or warrants medical evaluation.	Present

The reduction in quality of life among FND patients is well-documented, primarily due to delays in diagnosis following symptom onset [9]. These delays pose a public health challenge, emphasizing the urgent need for better diagnostic algorithms for patients showing signs of neurological events [1,2,10]. It’s essential to recognize that not every seizure-like event is caused by epilepsy [2,11]. Therefore, clinicians need to use validated diagnostic criteria, such as those outlined by LaFrance [6], to differentiate epilepsy from FND through careful analysis of event characteristics [1,11]. The proper use of VEEG, as demonstrated in this case, is crucial for accurate diagnosis and treatment [1,8,11].

Effective management of FND requires a structured plan. The six pillars proposed by Kanemoto [1] offer a helpful framework: accurate diagnosis, appropriate treatment, ongoing care for chronic FND, collaboration between neurology and psychiatry, personalized treatment plans, and thorough patient education about their condition [10,1]. In our patient's case, progress has been made in most areas. However, diagnosis was the most formidable challenge; now, follow-up care and the patient’s acceptance of her condition are paving the way for a more tailored and effective treatment plan.

A major overarching challenge was the stigma involved, both within the healthcare system and in the patient’s personal experience. The history of FND ties back to the concept of hysteria, a term filled with sexist, ambiguous, and pejorative meanings. Although hysteria was officially removed from diagnostic manuals, its symbolic legacy persists in clinical practice, often leading to perceptions of these symptoms as inexplicable, fabricated, or signs of personality flaws [5]. Researchers such as Trimble and Reynolds have described how this ambiguity has undermined the legitimacy of the diagnosis, thereby delaying recognition of FND as a valid and treatable condition [6].

Furthermore, stigma often damages trust between patients and healthcare providers. A recent systematic review by McLoughlin et al. [12] showed that patients with FND usually face systemic stigmatization, which negatively impacts treatment adherence, quality of life, and health outcomes [7]. In this case, providing a clear, empathetic explanation based on a biopsychosocial model was vital in helping the patient accept her diagnosis and engage in treatment.

A successful approach to FND calls for a shift in thinking from viewing it as an exclusion diagnosis to understanding it as a positive, explanatory condition. Hallett et al. advocate for seeing FND as a neurobiological dysfunction with shared mechanisms across different types, highlighting the need for specific clinical pathways, interdisciplinary care, and ongoing follow-up [9]. This case highlights the pressing need to enhance medical education, develop comprehensive diagnostic and therapeutic protocols, and foster a clinical environment free from stigma.

Throughout the patient's journey, progress has been made in most of these areas. However, diagnosis was the most significant challenge. With ongoing follow-up and her gradually accepting her diagnosis, a more effective and personalized treatment strategy is now within reach.

## Conclusions

Although FND remains a condition frequently subject to misunderstandings and misdiagnosis, its clinical manifestations often resemble those associated with structural neurological disorders, such as epilepsy, thereby resulting in unnecessary treatments and prolonged patient suffering. The early identification of FND, utilizing validated clinical criteria and diagnostic tools such as VEEG, is crucial for effective management. The integration of neurology, psychiatry, and psychotherapy, coupled with effective communication with patients, plays an indispensable role in promoting treatment adherence, mitigating iatrogenic harm, and facilitating functional recovery. Ultimately, a compassionate and comprehensive approach is essential for achieving improved outcomes for patients diagnosed with FND.

## Figures and Tables

**Figure 1:**
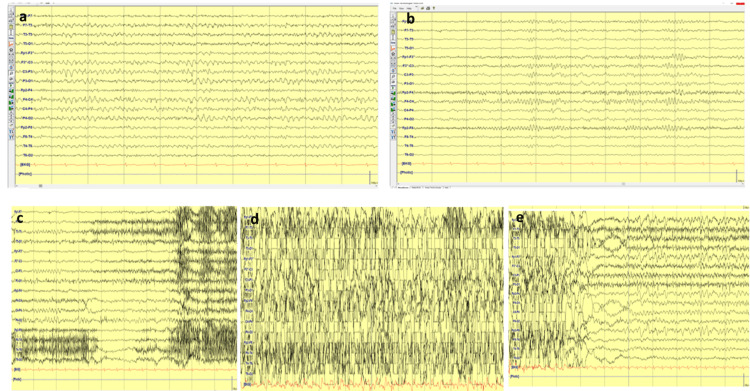
Video-EEG recording with non-epileptic paroxysmal events. Interictal EEG during wakefulness and N1–N2 sleep stages was normal. No epileptiform activity was observed. Six events were recorded during wakefulness (07:58:30, 09:03:52, 09:05:00, 09:08:30, 09:12:09, and 12:45:50). These events were characterized by fixed staring, blinking, apparent unresponsiveness to external stimuli, purposeful head movements, eye closure, clenched or clawlike hands, flexion of the upper limbs, extension of the lower limbs, sustained upward eye deviation, head turning to the right and left, generalized tremor with uncoordinated limb movements, and hyperventilation. At the end of the events, the patient obeyed commands, understood, and produced speech. None of the events showed an electrical correlate. Only muscle and movement artefacts were observed. The individual images are described as; (a–b) Interictal EEG during wakefulness and N1–N2 sleep. Normal background activity with preserved symmetry and sleep–wake transitions. No epileptiform abnormalities are observed. (c) Onset of the event. No ictal EEG changes are identified at the beginning of the clinical episode. (d) Event. EEG shows non-epileptic paroxysmal activity without ictal or epileptiform patterns. (e) End of the event. EEG returns to baseline activity with no postictal abnormalities.

## Appendices

Appendix A

A 6-hour video-EEG recording was performed during wakefulness and physiological sleep. The recording shows a baseline activity consisting of an alpha rhythm of 8-10 Hz with an amplitude of 20-40 μV, symmetric and synchronous, with an appropriate anteroposterior gradient. The patient reached N2 sleep stage.
